# Aggregation-Based Bacterial Separation with Gram-Positive Selectivity by Using a Benzoxaborole-Modified Dendrimer

**DOI:** 10.3390/molecules28041704

**Published:** 2023-02-10

**Authors:** Ayame Mikagi, Yotaro Takahashi, Nobuyuki Kanzawa, Yota Suzuki, Yuji Tsuchido, Takeshi Hashimoto, Takashi Hayashita

**Affiliations:** 1 Department of Materials and Life Sciences, Faculty of Science and Technology, Sophia University, 7-1 Kioi-cho, Chiyoda-ku, Tokyo 102-8554, Japan; 2 Department of Life Science and Medical Bioscience, School of Advanced Science and Engineering, Waseda University (TWIns), 2-2 Wakamatsu-cho, Shinjuku-ku, Tokyo 162-8480, Japan

**Keywords:** bacterial separation, benzoxaborole, dendrimer, Gram-positive bacteria, saccharide recognition

## Abstract

Antimicrobial-resistant (AMR) bacteria have become a critical global issue in recent years. The inefficacy of antimicrobial agents against AMR bacteria has led to increased difficulty in treating many infectious diseases. Analyses of the environmental distribution of bacteria are important for monitoring the AMR problem, and a rapid as well as viable pH- and temperature-independent bacterial separation method is required for collecting and concentrating bacteria from environmental samples. Thus, we aimed to develop a useful and selective bacterial separation method using a chemically synthesized nanoprobe. The metal-free benzoxaborole-based dendrimer probe BenzoB-PAMAM(+), which was synthesized from carboxy-benzoxaborole and a poly(amidoamine) (PAMAM) dendrimer, could help achieve Gram-positive bacterial separation by recognizing Gram-positive bacterial surfaces over a wide pH range, leading to the formation of large aggregations. The recognition site of benzoxaborole has a desirable high acidity and may therefore be responsible for the improved Gram-positive selectivity. The Gram-positive bacterial aggregation was then successfully collected by using a 10 μm membrane filter, with Gram-negative bacteria remaining in the filtrate solution. BenzoB-PAMAM(+) will thus be useful for application in biological analyses and could contribute to further investigations of bacterial distributions in environmental soil or water.

## 1. Introduction

The proliferation of antimicrobial-resistant (AMR) bacteria has become a critical global concern [1,2,3] from the standpoint of achieving the Sustainable Development Goals [4,5]. The World Health Organization (WHO) has indicated that AMR is one of the top 10 global public health threats facing humanity. Several drugs, such as antibiotics, which are essential in treating infectious diseases, promote the development of AMR bacteria. When exposed to antibiotics AMR bacteria survive longer than other bacteria, and genetic mutations are thus passed on to the following generations. The misuse or overuse of antimicrobial agents and medicines is a driving force for AMR because improper antibiotic use accelerates genetic mutations. New AMR bacterial strains no longer respond to antibiotics and other antimicrobial drugs, rendering infections increasingly difficult or impossible to treat, increasing the risk of disease, severe illness, and death. High numbers of antibiotic-resistant bacteria have already been observed globally, suggesting a decrease in the number of effective antibiotics, which will result in reduced efficacy in treating infections.

According to the Global Antimicrobial Resistance and Use Surveillance System (GLASS), the rate of resistance to ciprofloxacin, an antibiotic commonly used to treat urinary tract infections, varied from 8.4 to 92.9% for *Escherichia coli* [6]. This high resistance rate suggests that ciprofloxacin is now ineffective in over 50% of the population in many countries; however, AMR bacteria have been infecting humans, animals, plants, and the environment, and have spread worldwide. Therefore, investigating the bacteria that live in environments such as water [7,8] or soil [9,10] is crucial in monitoring the development and spread of AMR bacteria. Monitoring is also useful for solving other problems, such as agricultural pesticide resistance [11]. One study reported that the lack of countermeasures would result in global losses of approximately USD 100 trillion annually [12]. Neither the economic impacts nor the health problems associated with AMR can therefore be ignored [13].

Metagenomic analyses, which are one of the most powerful tools for the analysis of environmental samples, are often used to study microbiomes [14]. The methods used for the recognition and collection of environmental bacteria differ in terms of efficacy and are thus important in terms of monitoring. Since impurities, such as mud or other organisms, must be separated from samples, size exclusion filters are often used before bacterial analyses; however, because filters are not selective, impurities of the same or smaller diameter are not removed. Hence, bacterial collection methods that can selectively recognize bacteria are required.

The problem has attracted the interest of many researchers, with biologists and chemists attempting to develop novel recognition or separation methods. Biological analysis methods, such as an immunosensing [15,16], enzyme-linked immunosorbent assay [17] or the polymerase chain reaction [18,19,20], which have high sensitivity and are species-specific because of their biological properties, have all been utilized with the aim of selective bacterial recognition, along with other methods, such as surface-enhanced Raman spectroscopy [21,22] or electrochemical detection [23]. However, to the best of our knowledge, these methods require advanced technical skills and expensive instruments, and are thus unsuitable in many situations.

Extraction methods, such as polymer-based separations, have also been investigated worldwide [24,25]. Magnetic nanoprobes, magnetic beads that are labeled with specific bacterial recognition sites, are particularly useful for separating and collecting bacteria [26]. In this method, magnetic-nanoprobe-bound bacteria are attracted to a magnet placed near the sample tube. The solution is removed, and the bacteria are obtained with the magnetic probe. Research that developed both magnetic probes and specific devices has also been reported [27]. Biological materials, including immunogenic materials, are generally used to recognize and select bacteria under this method [28]; however, problems such as nanoprobe self-aggregation, the limited experimental conditions (such as pH and temperature) under which specific biological materials can be used, or the need for expensive reagents or devices, such as specific antibodies, mean that easier, more reasonable, and useful methods with which to recognize and collect bacteria are required.

**Figure 1:**
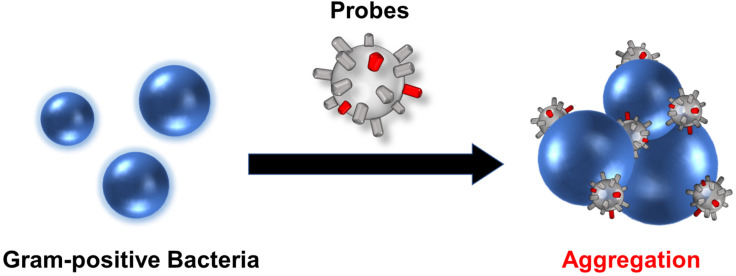
Schematic image of selective bacterial recognition by boronic acid-modified PAMAM dendrimer probes.

**Figure 2:**
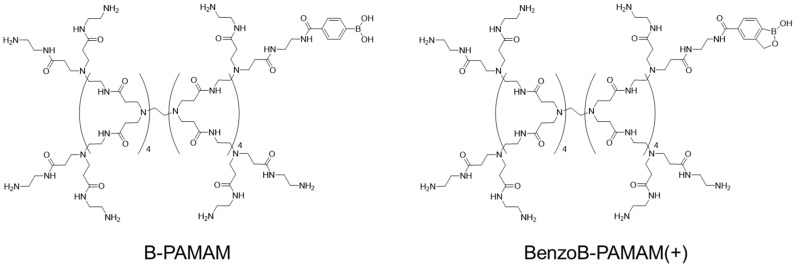
Structures of boronic acid-based PAMAM dendrimer probes.

Our research group is focused on separation by aggregation in addition to recognition by boronic acid compounds. In 2019 we reported a chemically modified poly(amidoamine) (PAMAM) dendrimer, which has a few phenylboronic acids on its terminal structure that could recognize and form aggregates of Gram-positive bacteria, such as *Staphylococcus aureus* (Figure 1 and Figure 2) [29], as confirmed by the naked eye. The core PAMAM dendrimers forming the nanoprobe, which comprise a group of synthetic spherical polymers [30], have desirable characteristics that allow bacterial extraction, water solubility, biocompatibility, and tolerance to self-aggregation, contrary to other nanoprobe cores, such as silica nanoparticles [31]. Boronic acid compounds are known as saccharide chemosensors [32,33] because they form boronate esters with the *cis*-diols in saccharides [34]. Boronic acid-based chemosensors are preferable for bacterial recognition because they are metal-free compounds that can rapidly recognize a target, such as bacterial saccharides, at room temperature (rt). We observed that our phenylboronic acid-modified PAMAM dendrimer (B-PAMAM) recognized lipoteichoic acid (LTA) [35], which is a surface structure that is specific to Gram-positive bacteria [36] and includes *cis*-diols [37]. B-PAMAM recognized LTA saccharides with the help of an electrostatic interaction between the positively charged amino terminus of B-PAMAM and the bacterial anionic surface [35,38]; however, the recognition ability was insufficient for obtaining selective aggregation and succeeding in bacterial separation. A turbidity measurement proved that only approximately half of the bacteria in the suspension were included in the aggregation owing to B-PAMAM exposure [29].

Therefore, in this study another boronic acid modification with stronger recognition ability is required to improve the selectivity and yield of Gram-positive bacteria that can be obtained using this method. Based on these requirements, carboxy-benzoxaborole, which should have a stronger affinity for saccharides rather than carboxy-phenylboronic acid, was prepared [39]. The improved benzoxaborole-modified PAMAM dendrimer, BenzoB-PAMAM(+) (Figure 2), was then used to investigate bacterial selectivity and pH tolerance, as well as its use in further applications.

## 2. Results and Discussion

### 2.1. Characteristics of the Boronic Acid-Based BenzoB-PAMAMs Nanoprobes

#### 2.1.1. Structure and Synthesis of BenzoB-PAMAMs

**Scheme 1:**
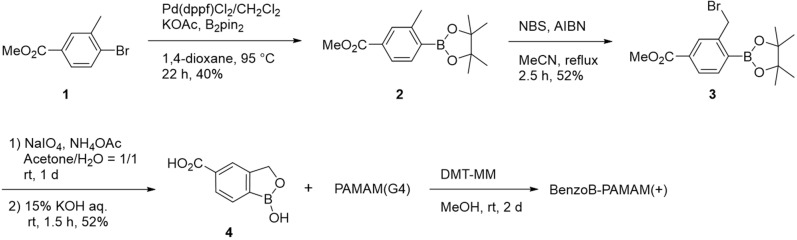
Synthesis of the benzoxaborole segment and BenzoB-PAMAM(+).

**Scheme 2:**
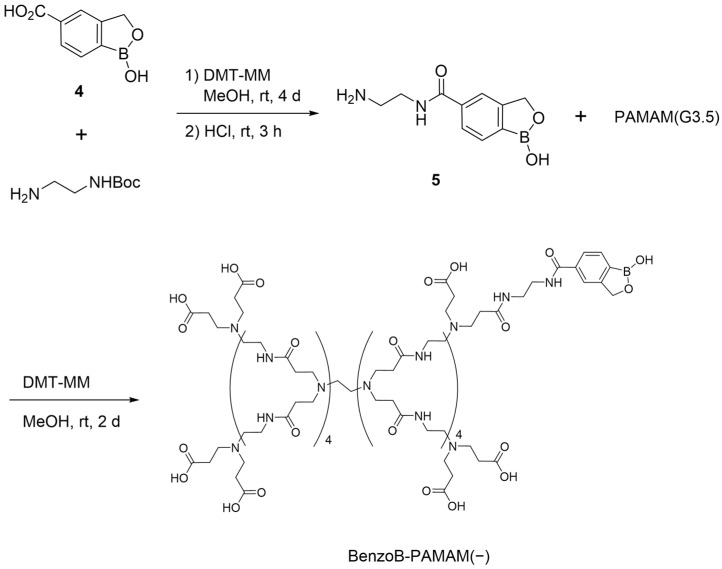
Synthesis of BenzoB-PAMAM(−).

First, we focused on benzoxaborole-modified PAMAM dendrimers (BenzoB-PAMAMs) instead of B-PAMAM. Since saccharide recognition, which results from the formation of boronate esters between boronic acids and *cis*-diols, mainly proceeds from conjugate tetrahedral boronate anions, boronic acids with large acid dissociation constants (*K*_a_s) are preferred. Benzoxaborole is a boronic acid analog known for its low p*K*_a_ value. For instance, the p*K*_a_ value of phenylboronic acid is approximately 8.9 [40], whereas that of benzoxaborole is 7.5 [41]. BenzoB-PAMAM might thus have a stronger affinity and better pH tolerance than B-PAMAM. The 4-carboxy-benzoxaborole segment was then synthesized and subjected to condensation with the amine-terminated PAMAM(G4) dendrimer (Scheme 1) to form BenzoB-PAMAM(+). Anionic BenzoB-PAMAM(−) was synthesized from the carboxylic acid-terminated PAMAM(G3.5) dendrimer for comparison with BenzoB-PAMAM(+) (Scheme 2) in the same manner as BenzoB-PAMAM(+). The amine-modified segment **5** was synthesized from a carboxy-benzoxaborole segment and *N*-Boc-diaminoethane (4-amino-benzoxaborole was not used) to produce a molecule with the same p*K*_a_ value as BenzoB-PAMAM(+).

#### 2.1.2. Surface Properties of BenzoB-PAMAMs

**Figure 3:**
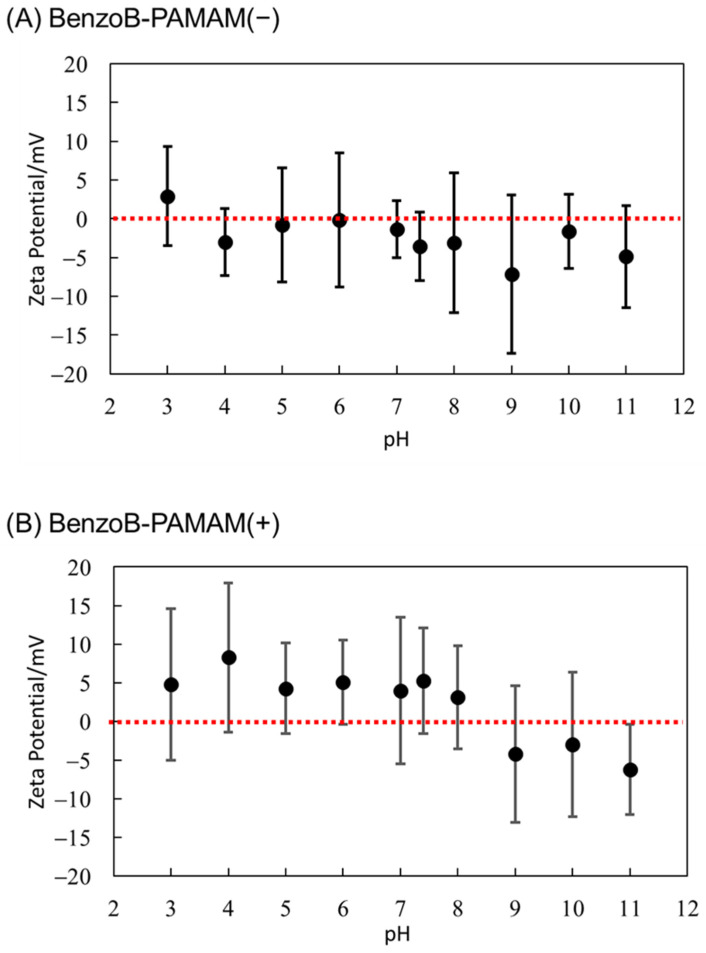
Zeta potential measurement of BenzoB-PAMAMs in PBS (*n* = 5). [probe] = 6.6 × 10^−6^ M. Bars express standard deviations (SDs) from five independent experiments. (**A**) BenzoB-PAMAM(−). (**B**) BenzoB-PAMAM(+).

The zeta potential was measured to estimate the electrostatic interaction between BenzoB-PAMAMs and the bacterial surface (Figure 3). We confirmed that the BenzoB-PAMAMs were successfully synthesized and that the desired charges were obtained by using the PAMAM dendrimer cores. BenzoB-PAMAM(+), with an amine terminus, is positively charged (Figure 3A), whereas BenzoB-PAMAM(−), with a carboxylic acid terminus, shows a relatively anionic surface (Figure 3B). The results obtained by using BenzoB-PAMAM(+) indicate that the zeta potential changed from positive to negative, while the pH increased from 8 to 9. This change may result from either the terminal primary amines or benzoxaborole modification. The assignment is further discussed below. As bacteria have negatively charged surfaces, the attraction effect of BenzoB-PAMAM(+) was likely electrostatic. In contrast to BenzoB-PAMAM(+), the negatively charged BenzoB-PAMAM(−) might be affected by electrostatic repulsion.

### 2.2. Bacterial Recognition by BenzoB-PAMAMs

#### 2.2.1. Recognition Confirmed by a Turbidity Measurement and Direct Observation

**Figure 4:**
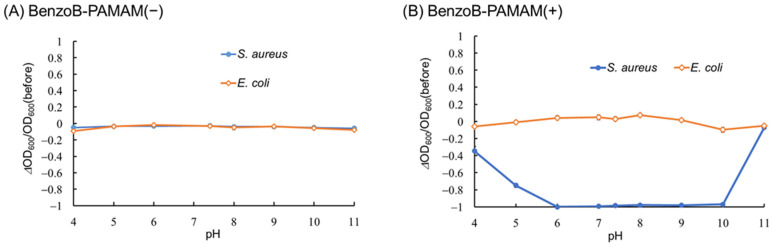
Turbidity changes in bacterial suspensions containing BenzoB-PAMAMs in PBS (*n* = 3). [BenzoB-PAMAM(−) or BenzoB-PAMAM(+)] = 3.3 × 10^−6^ M. [*S. aureus* IAM1011 or *E. coli* K12W3110] = 2.3 × 10^8^ CFU·mL^−1^. Bars represent the SD in three separate experiments. (**A**) BenzoB-PAMAM(−). (**B**) BenzoB-PAMAM(+).

**Figure 5:**
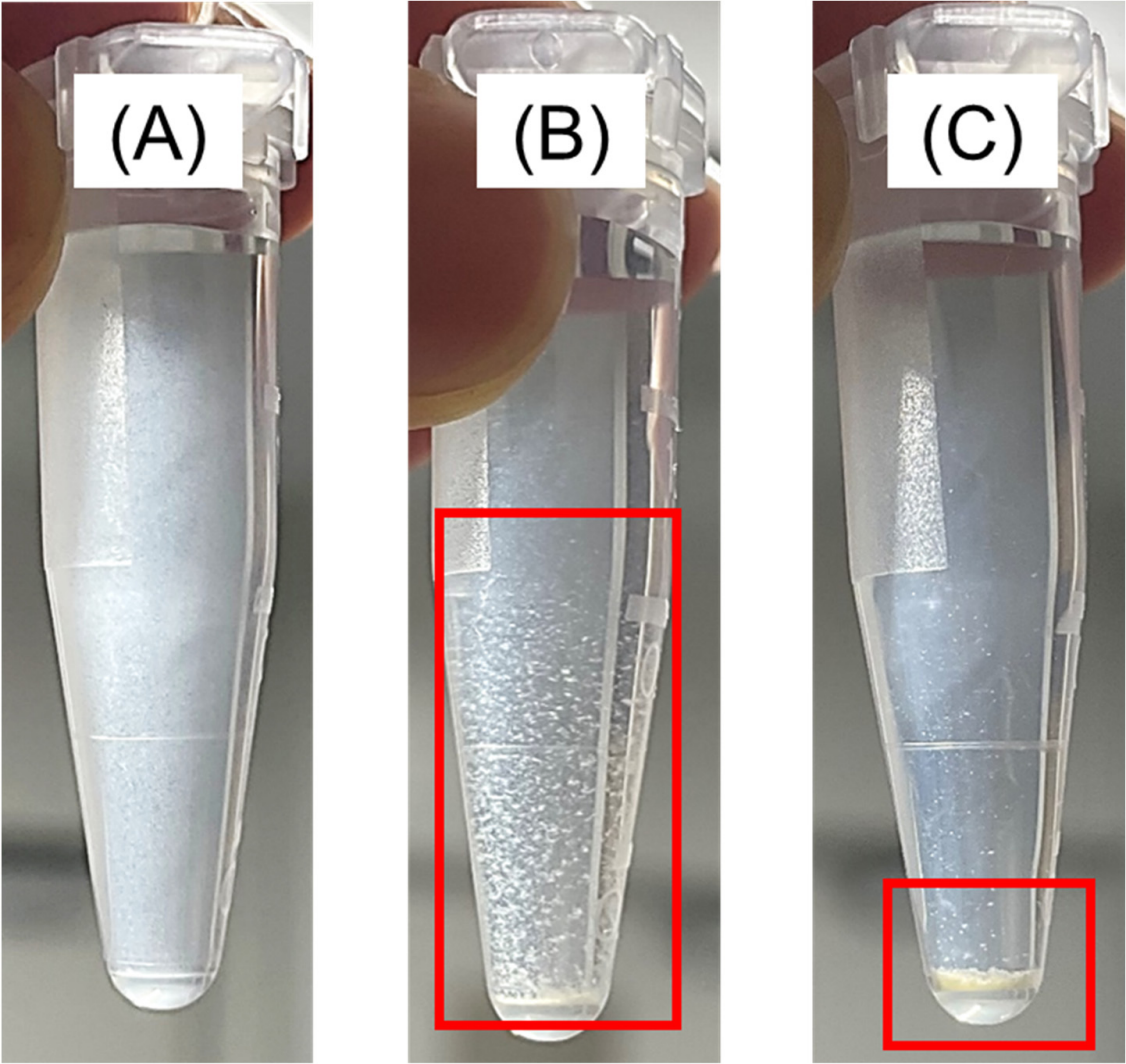
Aggregate formation for BenzoB-PAMAM(+) in PBS. [BenzoB-PAMAM(+)] = 3.3 × 10^−6^ M, [*S. aureus* IAM1011] = 2.3 × 10^8^ CFU·mL^−1^; aggregates are highlighted using red squares. (**A**) pH of 4.0; (**B**) pH of 5.0; and (**C**) pH of 6.0.

Turbidity was measured (Figure 4) to elucidate the effects of electrostatic interaction on bacterial recognition. When a probe recognizes bacterial saccharides, the complexes formed by bacteria and the probe should result in changes to the turbidity, as has been reported previously [29]. Measurements were recorded between pHs of 4.0 and 11.0 to cover all biologically relevant pH conditions. Researchers have reported the growth of Gram-positive bacteria, *S. aureus*, in environments with pHs of 4.0−9.8 [42] and Gram-negative bacteria, *E. coli*, at pHs of 4.5−9.0 [43]. The results in Figure 4A indicate that turbidity does not change under all pH conditions for BenzoB-PAMAM(−). As *S. aureus* [44,45] and *E. coli* [46,47] have negatively charged surfaces, in the same manner as general bacteria, the results indicate that electrostatic repulsion between the anionic terminus of BenzoB-PAMAM(−) and the bacterial surface disturbed any saccharide recognition. In contrast, BenzoB-PAMAM(+) selectively recognized Gram-positive *S. aureus* under wide pH conditions, as seen in Figure 4B (from a pH of 5 or 6 to 10). We have already reported that electrostatic interaction between cationic dendrimers and negatively charged bacterial surfaces enhanced recognition ability, whereas electrostatic repulsion disturbed bacterial recognition [35,38]. The resulting aggregation was easily confirmed by the naked eye (Figure 5). Images revealed no aggregation at a pH of 4.0 (Figure 5A); however, it is visible at pHs of 5.0 (Figure 5B) and 6.0 (Figure 5C), as suggested by the results of the turbidity measurements. Considering the previous results, that phenylboronic acid-modified B-PAMAM led to selectivity from a pH of 6−8 or 7−9 [38], BenzoB-PAMAM(+) has a significantly improved recognition ability compared with that of B-PAMAM. The results suggest that the lower p*K*_a_ value of boronic acid results in a stronger recognition force towards the LTA of Gram-positive bacteria.

**Figure 6:**
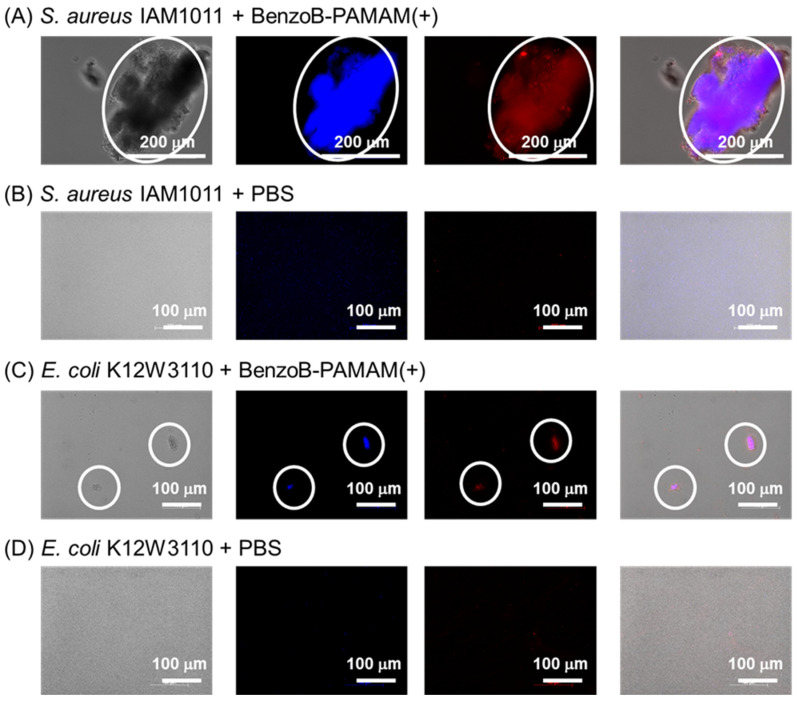
Microscope observation of *S. aureus* IAM1011 and *E. coli* K12W3110 at a pH of 7.4, adjusted with PBS. [BenzoB-PAMAM(+)] = 3.3 × 10^−6^ M or none; bacterial concentration was set at 2.3 × 10^8^ CFU·mL^−1^ for *S. aureus* IAM1011 and 5.0 × 10^8^ CFU·mL^−1^ for *E. coli* K12W3110. From left to right: DIC, DAPI, PI, and merged microscopy images. Scale bar = 200 µm in (**A**) and 100 µm in (**B**–**D**).

Aggregation was also confirmed through microscopic observation to obtain further information (Figure 6). Compared with bacterial images (Figure 6B,D), images depicting BenzoB-PAMAM(+) suspensions revealed complexes resulting from the interaction between bacteria and BenzoB-PAMAM(+) (Figure 6A,C); however, the aggregation size is far more apparent for Gram-positive *S. aureus* and Gram-negative *E. coli*. We considered that the aggregation size was instrumental in changes in turbidity and the presence of visible precipitation. Although the *E. coli* suspension formed minute aggregations with BenzoB-PAMAM(+), no structure was observed at the bottom of the sample tube. We concluded that the aggregation could be extracted via scooping. Notably, some aggregated bacteria appeared dead, as we have previously reported [48]; however, this was not a problem because metagenomic analyses, which are conducted after bacterial extraction, are not affected by the state of bacteria.

#### 2.2.2. Improved Recognition in Association with the Desirable p*K*_a_ Value of Benzoxaborole

**Figure 7:**
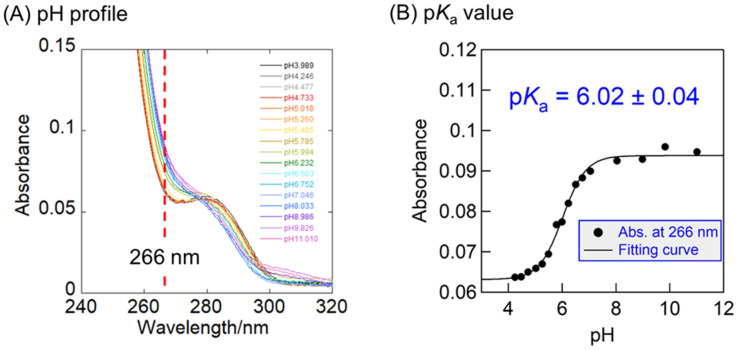
UV–Vis absorbance results for benzoxaborole **5** in PBS from pHs of 4.0 to 11.0. [compound **5**] = 10.0 × 10^−6^ M. (**A**) UV–Vis absorption spectra under various pH conditions. (**B**) Summary at 266 nm from which the p*K*_a_ value was calculated.

The p*K*_a_ value of the benzoxaborole recognition sites on BenzoB-PAMAM(+) was investigated using benzoxaborole with an amine terminal. UV–Vis absorption spectra were measured between pHs of 4.0 and 11.0 (Figure 7A). Absorbance at 266 nm, which produces large differences under different pH conditions, was used to determine the p*K*_a_ value (Figure 7B). By the inflexion point, a p*K*_a_ value of 6.0 was observed for the benzoxaborole comprising BenzoB-PAMAM(+). This value is consistent with the results of the turbidity measurements, with the probe recognizing bacteria at pHs of 5−6. Benzoxaborole can easily exist as a tetrahedral anion because of its low p*K*_a_ value, and its character is desirable for saccharide recognition, which starts preferentially in the initial boronate anions. It should be noted that the change in the zeta potential at pHs around 8−9 was not caused by benzoxaborole, according to its p*K*_a_ value of 6.0, and may have been caused by the terminal primary amines on BenzoB-PAMAM(+). Researchers have reported p*K*_a_ values of 8−9 for the terminal primary amines on PAMAM(G4) dendrimers [49], matching the pH range, which shows changes in line with the zeta potential at pHs of 8−9 (Figure 3B). In summary, BenzoB-PAMAM(+) had the desired low p*K*_a_ value, which enhanced selective bacterial recognition, and the zeta potential of the probe was mainly determined by terminal amine groups rather than the benzoxaborole recognition site.

#### 2.2.3. Bacterial Selectivity Using BenzoB-PAMAM(+)

**Figure 8:**
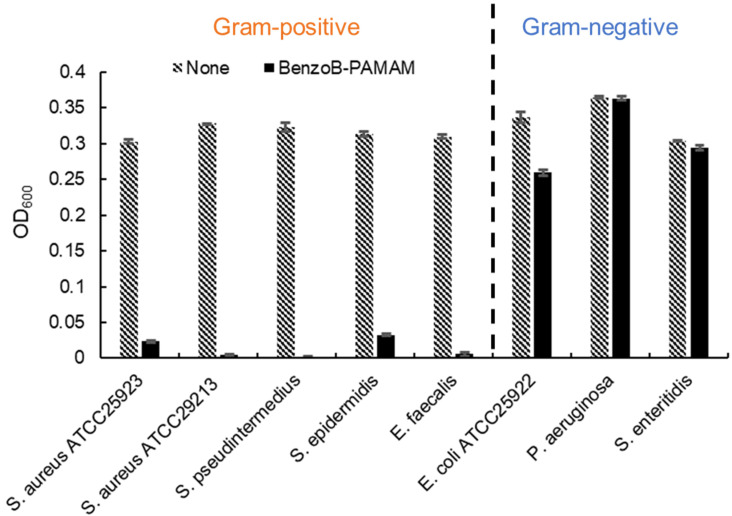
Turbidity experiments using various bacteria at a pH of 7.4, adjusted with PBS (*n* = 3). [BenzoB-PAMAM(+)] = none or 3.3 × 10^−6^ M; bacterial concentration was set to OD_600_ = 0.3. Bars represent SDs from three independent experiments.

Changes in the turbidity were also measured using eight different bacteria to investigate bacterial selectivity (Figure 8). The results indicate that BenzoB-PAMAM selectively recognized Gram-positive bacteria, with a decrease in turbidity. Compared with the results obtained using B-PAMAM, which showed a decrease of approximately 50% in turbidity for a Gram-positive bacterial suspension [29], a decrease of almost 100% for BenzoB-PAMAM(+), with its low p*K*_a_ value, indicates its suitability for bacterial separation. A turbidity of approximately zero means that almost all of the bacteria in a suspension have formed aggregations with probes and can be extracted with a sufficient yield. We confirmed that the improved recognition and Gram-positive selectivity were derived from LTA recognition (Figures S1 and S2).

**Figure 9:**
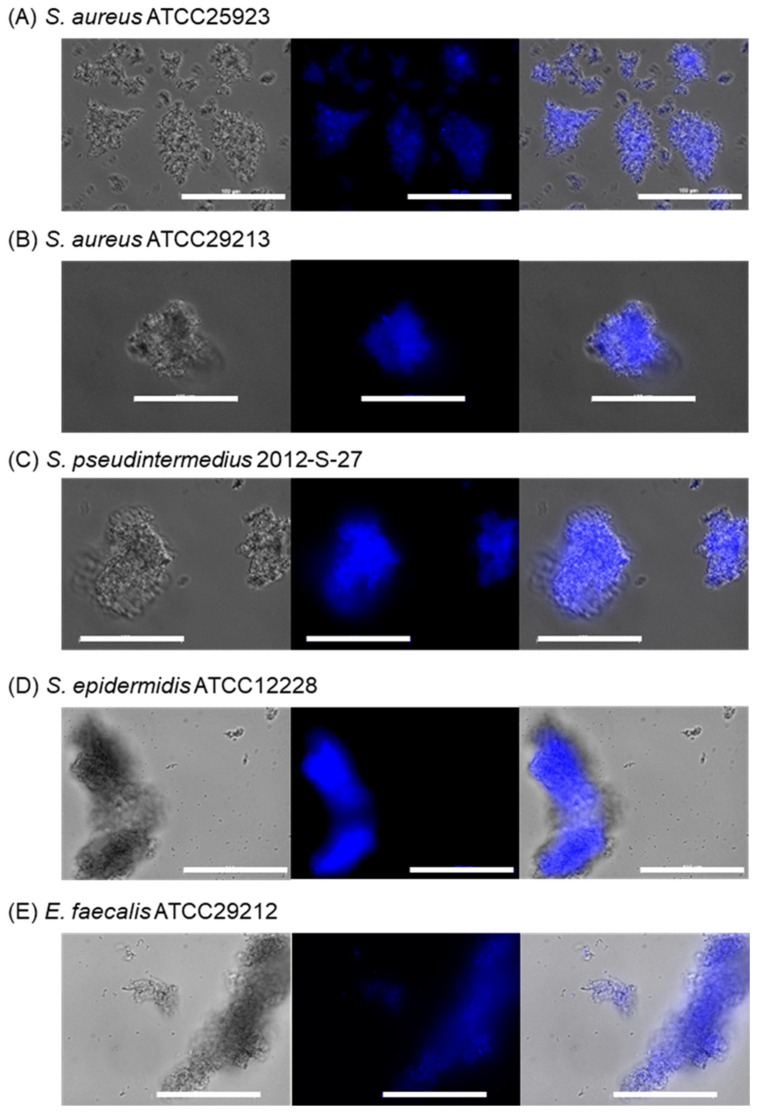
Microscope images of Gram-positive bacteria BenzoB-PAMAM(+) at a pH of 7.4, adjusted with PBS. [BenzoB-PAMAM(+)] = 3.3 × 10^−6^ M; bacterial concentration was set at OD_600_ = 0.3. From left to right: DIC, DAPI, and merged microscopy images. Scale bar = 100 µm. (**A**) *S. aureus* ATCC25923. (**B**) *S. aureus* ATCC29213. (**C**) *S. pseudintermedius* 2012-S-27. (**D**) *S. epidermidis* ATCC12228. (**E**) *E. faecalis* ATCC29212.

**Figure 10:**
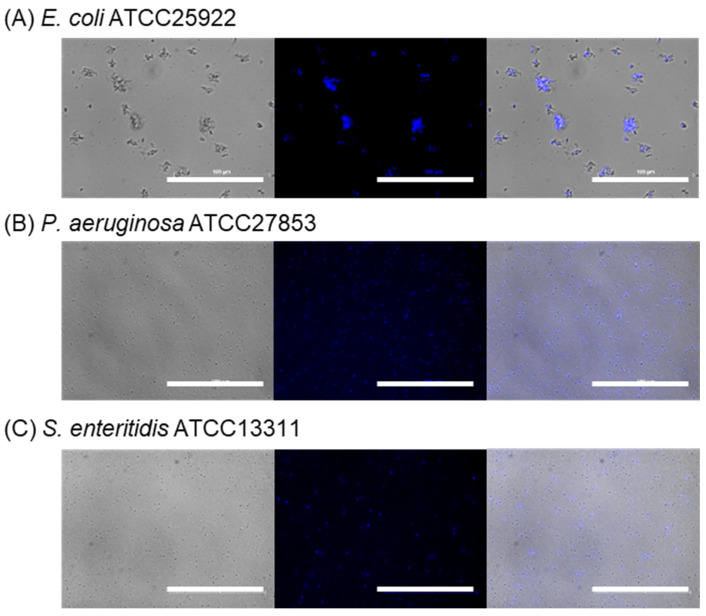
Microscope images of Gram-negative bacteria obtained using BenzoB-PAMAM(+) at a pH of 7.4, adjusted with PBS. [BenzoB-PAMAM(+)] = 3.3 × 10^−6^ M; bacterial concentration was set at OD_600_ = 0.3. From left to right: DIC, DAPI, and merged microscopy images. Scale bar = 100 µm. (**A**) *E. coli* ATCC25922. (**B**) *P. aeruginosa* ATCC27853. (**C**) *S. enteritidis* ATCC13311.

From the perspective of bacterial extraction by aggregation, the size of an aggregation is significant. Typically, Gram-positive bacterial aggregates that are significantly larger than bacteria (or some minute aggregation of Gram-negative bacteria) could be obtained by scooping up the precipitation followed by washing through a membrane filter of an appropriate pore size. Eight bacteria used in Figure 8 were observed via microscopy to confirm the size of the aggregations (Figure 9 and Figure 10). The microscopy images demonstrate that Gram-positive bacteria, which result in dramatic turbidity decreases, formed large aggregates of approximately 50−200 μm (Figure 9). We also confirmed that a low concentration of bacteria (10^6^ CFU·mL^−1^) formed approximately 10 μm aggregates (Figure S3). Gram-negative bacteria did not produce such large aggregates, even *E. coli* ATCC25922, which formed minute aggregates (Figure 10). As the aggregation size of *E. coli* ATCC25922 was smaller than 10 μm, we concluded that a membrane with a filter of a 10 μm pore size was suitable for extracting and washing Gram-positive bacterial aggregations by trapping Gram-positive bacteria while allowing Gram-negative bacteria to pass through.

#### 2.2.4. Filtration for Separating Aggregations

**Figure 11:**
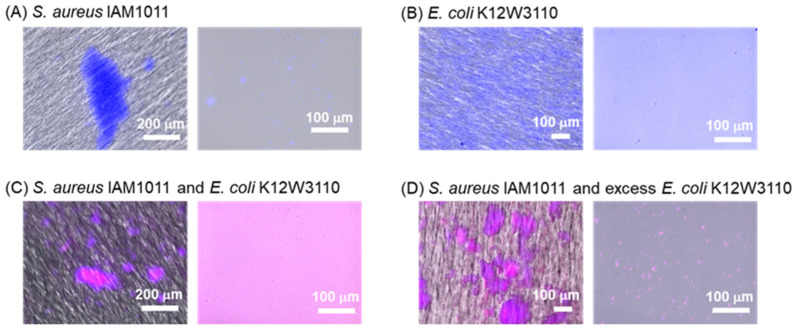
Microscope images of filter and filtrate using BenzoB-PAMAM(+) at a pH of 7.4, adjusted with PBS. [BenzoB-PAMAM(+)] = 3.3 × 10^−6^ M. From left to right: merged microscopy images of filter and filtrate samples. (**A**) 1.4 × 10^8^ CFU·mL^−1^ of *S. aureus* IAM1011 stained with DAPI. (**B**) 3.0 × 10^8^ CFU·mL^−1^ *E. coli* K12W3110 stained with DAPI. (**C**) 1.0 × 10^8^ CFU·mL^−1^ *S. aureus* IAM1011 (DAPI) and *E. coli* K12W3110 (EB). (**D**) 1.4 × 10^8^ CFU·mL^−1^ *S. aureus* IAM1011 (DAPI) and 3.0 × 10^8^ CFU·mL^−1^ *E. coli* K12W3110 (EB).

We attempted to separate the Gram-positive bacterial aggregates from the probes by filtration with a 10 µm pore size membrane filter. The obtained materials on the filter and in the filtrate were inspected separately by using a microscope (Figure 11). Suspensions that include *S. aureus* IAM1011 typically exhibit large aggregations on the filters (Figure 11A,C,D). The sample without *S. aureus* IAM1011 revealed bacteria in the filtrate solution with no aggregation on the filter (Figure 11B). These results show that the membrane filter could separate the aggregation by *S. aureus* IAM1011 from the sample solution as desired. Although the aggregation contained a certain amount of *E. coli* K12W3110 stained with EB (Figure 11C,D), *E. coli* K12W3110 itself did not aggregate, as seen in Figure 11B. We also confirmed that excess *E. coli* K12W3110 did not disturb the aggregation process (Figure 11D). Concordantly, *E. coli* K12W3110 was observed in the filtrate solution, meaning that only a small number of *E. coli* were involved in the aggregation. Another bacterial mixture, comprising *S. aureus* ATCC25923 and *E. coli* ATCC25922, also exhibited aggregation on the filter and dispersed bacteria in the filtrate solution (Figure S4). In summary, Gram-positive bacteria were successfully separated by filtering the aggregates through a membrane filter with a 10 µm pore size.

## 3. Materials and Methods

### 3.1. Reagents and Apparatus

#### 3.1.1. Chemical Reagents

All reagents and solvents were purchased from commercial suppliers and used without further purification unless otherwise specified. Acetone, super dehydrated (016-23465), agar (018-15811), ammonium acetate (NH_4_OAc, 016-02845), *n*-bromosuccinimide (NBS, 021-07232), dichloromethane (DCM, 139-02444),1,4-dioxane, super dehydrated (040-31651), ethyl acetate (EtOAc, 051-00351), hydrochloric acid (080-01066), magnesium sulfate, anhydrous (137-12335), potassium acetate (KOAc, 166-01372), 30 % potassium hydroxide solution (32904-00), sodium chloride (191-01665), sodium periodate (197-02402), and sodium sulfate (197-03345) were purchased from the FUJIFILM Wako Pure Chemical Corporation (Osaka, Japan). 4′,6-Diamidino-2-phenylindole dihydrochloride *n*-hydrate (DAPI, D523), ethidium bromide solution (EB, BS06), and propidium iodide solution (PI, P378) were purchased from DOJINDO LABORATORIES (Kumamoto, Japan). 2,2′-azobis(isobutyronitrile) (AIBN, A0566), [1,1′-bis(diphenylphosphino) ferrocene]palladium(II) dichloride dichloromethane adduct (Pd(dppf)Cl_2_/CH_2_Cl_2_, B2064), bis(pinacolato)diboron (B_2_pin_2_, B1964), 4-(4,6-dimethoxy-1,3,5-triazin-2-yl)-4-methylmorpholinium chloride (DMT-MM, D2919), methyl 4-bromo-3-methylbenzoate (**1**, B2256), and *N*-(tert-butoxycarbonyl)-1,2-diaminoethane (*N*-Boc-diaminoethane, A1371) were purchased from the Tokyo Chemical Industry Co. Ltd. (Tokyo, Japan). Acetonitrile dehydrated (MeCN, 01837-95), dimethyl sulfoxide-d_6_ (DMSO-d_6_, 11560-33), methanol (MeOH, 25183-70), methanol-d_4_ (99.8 atom%D, 25183-70), and deuterium water (D_2_O, 99.8 atom%D, 10086-23) were purchased from the Kanto Chemical Co. Inc. (Tokyo, Japan). Omnipore membrane 10 μm × Φ13 mm (JCWP01300), poly(amidoamine) (PAMAM) dendrimer, ethylenediamine core, generation 4.0 (412449) solution, PAMAM dendrimer, ethylenediamine core, generation 3.5 solution, 10 wt.% in methanol (412430), and Swinnex Filter Holder Φ13 mm (SX0001300) were purchased from the Sigma-Aldrich Japan Co. LLC. (Tokyo, Japan). Bacto yeast extract (212750) and Bacto Tryptone (211705) were purchased from Nippon Becton, Dickinson Co. Ltd. (Tokyo, Japan). Phosphate-buffered saline (PBS, 2101) was purchased from the Cell Science & Technology Institute Inc. (Miyagi, Japan). Water was distilled and deionized twice by using a Milli-Q water system (WG222, Yamato Scientific Co. Ltd., Tokyo, Japan and Milli-Q Advantage, Merck Millipore, Burlington, MA, USA) before use. A Spectra/Por 6 dialysis bag (MW cutoff = 1000) was purchased from the Repligen Co. (Waltham, MA, USA).

#### 3.1.2. Apparatus

^1^H and ^13^C nuclear magnetic resonance (NMR) spectra were recorded on a JEOL JNM-ECA 500 spectrometer (500 MHz) by JEOL (Tokyo, Japan) at 300 K or a Bruker Avance III HD 400 MHz by Bruker (Billerica, MA, USA) at 300 K using a deuterated solvent. All pH values were recorded by using Horiba F-52 and F-72 pH meters (Horiba, Ltd., Kyoto, Japan). Ultraviolet−visible (UV−Vis) absorption spectra were measured by using a JASCO V-570 or V-760 UV−Vis spectrophotometer (JASCO Co., Tokyo, Japan) equipped with a Peltier Thermo controller and a 10 mm quartz cell. Zeta potential measurements were carried out at 25 °C by using a Zetasizer Nano ZS (Malvern Instruments Ltd., Malvern, Worcestershire, U.K.). Samples were shaken by using a MULTI SHAKER MS-300 (AS ONE Co., Osaka, Japan). Centrifugation was conducted by using a CF15RN (Hitachi High-Technologies, Co., Tokyo, Japan). The p*K*_a_ value was determined by using Igor Pro v5.0.3.0 (Wavemetrics inc., Portland, OR, USA) based on the acid dissociation model of monobasic acids.

### 3.2. Preparation of Dendrimer Probes

#### 3.2.1. Synthesis of Methyl 3-Methyl-4-(4,4,5,5-Tetramethyl-1,3,2-Dioxaborolan-2-Yl)Benzoate (**2**)

Compound **2** was synthesized by using commercially available compound **1** [39] under an argon atmosphere. Bis(pinacolato)diboron (1.1 eq, 1.68 g, and 6.6 mmol), KOAc (3.1 eq, 1.85 g, and 19 mmol), and Pd(dppf)Cl_2_/CH_2_Cl_2_ (2 mol%, 0.11 g, and 0.14 mmol) were added to the solution produced by dissolving compound **1** (1.0 eq, 1.38 g, and 6.0 mmol) in 1,4-dioxane (10.0 mL). The resulting orange reaction solution was stirred vigorously at 95 °C for 22 h before undesired precipitates were removed through filtration. The filtrate was then evaporated, and the product was extracted three times with DCM/water. The combined organic layers were dried over MgSO_4_ and concentrated in vacuo. Purification by silica gel column chromatography (DCM 100%) was then used to generate product **2** as a yellow oil (684 mg, 2.5 mmol, and 40%). The structure was confirmed from the resulting ^1^H NMR spectrum (Figure S5).

#### 3.2.2. Synthesis of Methyl 3-(Bromomethyl)-4-(4,4,5,5-Tetramethyl-1,3,2-Dioxaborolan-2-Yl)Benzoate (**3**)

Compound **3** was synthesized by using compound **2** under an argon atmosphere [39]. NBS (1.3 eq, 695 mg, and 3.9 mmol) and AIBN (2 mol%, 10 mg, and 0.06 mmol) were added to the solution generated by dissolving compound **2** (1.0 eq, 861 mg, and 3.1 mmol) in MeCN (15.0 mL). The solution was stirred at 90 °C for 2.5 h before the solvent was evaporated. DCM was added to the concentrated solution to remove the undesired white precipitation. Purification by silica gel column chromatography (DCM 100%) generated product **3** in the form of a yellowish oil (580 mg, 1.6 mmol, and 52%). The structure was confirmed by using the resulting ^1^H NMR spectrum (Figure S6).

#### 3.2.3. Synthesis of 1-Hydroxy-1,3-Dihydro-2,1-Benzoxaborole-5-Carboxylic Acid (**4**)

Compound **4** was synthesized by using compound **3** [39], which (1.0 eq, 580 mg, and 1.6 mmol) was dissolved in an acetone/water mixture at 1/1 (*v*/*v*). NaIO_4_ (4.9 eq, 1.74 g, and 8.1 mmol) and NH_4_OAc (5.0 eq, 630 mg, and 8.1 mmol) were added to the produced solution, and the mixture was stirred at rt for 1 day. The solvent was then evaporated, and the product was extracted three times with EtOAc/water. The combined organic layers were then dried over MgSO_4_ and concentrated in vacuo. Afterwards, 15% KOH aq. (3.0 mL) was added to the obtained product and stirred for 1.5 h at rt, followed by acidification via HCl aq. The obtained white precipitation, compound **4** (152 mg, 0.83 mmol, and 52%), was extracted by filtration, and the structure was confirmed by using the resulting ^1^H NMR spectrum (Figure S7).

#### 3.2.4. Synthesis of BenzoB-PAMAM(+)

Compound **4** (8.0 eq, 11.3 mg, and 64 μmol) and DMT-MM (40.0 eq, 88.2 mg, and 0.32 mmol) were dissolved in methanol (10.0 mL), and the reaction mixture was stirred at rt for 30 min [35]. The PAMAM(G4) dendrimer (1.4 mL, 1.0 eq, and 8.0 μmol) was added, and the reaction mixture was stirred at rt for 2 days. The reaction mixture was transferred into a Spectra/Por 6 dialysis bag and dialyzed against methanol as well as distilled water for several days before the purified product was lyophilized to produce white flocks (124.5 mg), the chemical structure of which was confirmed by a ^1^H NMR measurement (Figure S8). The number of benzoxaborole substituents was estimated from the corresponding peak area in the ^1^H NMR spectrum.

#### 3.2.5. Synthesis of *N*-(2-Aminoethyl)-1-Hydroxy-1,3-Dihydro-2,1-Benzoxaborole-5-Carboxamide (**5**)

*N*-Boc-diaminoethane (96 mg, 1.2 eq, and 0.6 mmol) and synthetic product **4** (89 mg, 1.0 eq, and 0.5 mmol) were dissolved in methanol (3 mL). The mixture was stirred at rt for 30 min [35]. DMT-MM (550 mg, 4.0 eq, and 2.0 mmol) was added to the solution, and a condensation reaction was performed at rt for 4 days. The product of the reaction was extracted three times with EtOAc, washed with brine, and dried over Na_2_SO_4_. The solvent was removed by evaporation, and HCl in methanol (4 M) was added to the residue. The solution was stirred at rt for 3 h to induce deprotection. The solvent was evaporated, and the final product **5** was obtained as a white solid (212 mg). The undesired byproducts were excluded by dialysis in the following synthesis. The identity was confirmed by ESI-HRMS spectral measurement and the resulting ^1^H and ^13^C NMR (Figures S9 and S10) spectra. The ESI-HRMS (*m*/*z*) calculated for C_11_H_16_B_1_N_2_O_3_ [M + CH_3_]^+^ 235.1254 found a figure of 235.1277.

#### 3.2.6. Synthesis of BenzoB-PAMAM(−)

Compound **5** (8.0 eq, 11.7 mg, and 49.5 μmol) and DMT-MM (32.0 eq, 54.6 mg, and 0.20 mmol) were dissolved in methanol (5.0 mL), and the reaction mixture was stirred at rt for 30 min [35]. The PAMAM(G3.5) dendrimer (1.0 mL, 1.0 eq, and 6.2 μmol) was added, and the reaction mixture was stirred at rt for 2 days. The reaction mixture was then transferred into a Spectra/Por 6 dialysis bag and dialyzed against methanol as well as distilled water for several days. The purified product was lyophilized to give white flocks (62.2 mg), the chemical structure of which was confirmed by a ^1^H NMR measurement (Figure S11). The number of benzoxaborole substituents was estimated from the corresponding peak area in the ^1^H NMR spectrum.

### 3.3. Biological Experiments

#### 3.3.1. Bacterial Culture

The lysogeny broth (LB) was composed of 2 g of Bacto Tryptone, 1 g of Bacto yeast extract, and 2 g of NaCl in 200 mL of distilled water. *S. aureus* IAM1011, *S. aureus* ATCC25923, *S. aureus* ATCC29213, *S. pseudintermedius* 2012-S-27, *S. epidermidis* ATCC12228, *Enterococcus faecalis* ATCC29212, *E. coli* K12W3110, *E. coli* ATCC25922, *Pseudomonas aeruginosa* ATCC27853, and *Salmonella enteritidis* ATCC13311 were provided by RIKEN BRC (Ibaraki, Japan). All bacteria were cultured at 37 °C overnight on LB agar plates. A mixture of 200 mL of LB and 3 g of agar was used to prepare each LB plate. Cultured colonies were selected and isolated overnight in LB at 37 °C. The suspension was centrifuged (10,000 rpm, 1 min) and washed twice with distilled water. The corresponding buffer was added to the washed cells, and the bacterial suspension was centrifuged. The concentration of the bacterial suspension was adjusted by measuring the optical density at 600 nm (OD_600_) after vortex mixing. The 4.5 × 10^8^ CFU·mL^−1^ *S. aureus* IAM1011 suspension gave OD_600_ = 1.0, and the 1.0 × 10^9^ CFU·mL^−1^ *E. coli* K12W3110 suspension gave OD_600_ = 1.0. The generated bacterial cultures were used in the following experiments.

#### 3.3.2. Bacterial Recognition

PAMAM dendrimer probes (0.75 mL, 6.6 × 10^−6^ CFU·mL^−1^) and bacterial cells in a buffer solution (0.75 mL, 4.5 × 10^8^ CFU·mL^−1^ unless otherwise noted) were mixed, and the OD_600_ was measured as a reference. Mixing was performed for 10 min at 2000 rpm. A turbidity measurement or fluorescence microscopy observation was conducted after standing for 10 min. For fluorescence microscopy, the DAPI solution was mixed with cultured bacteria in PBS, and excess dye was carefully washed off before mixing for 10 min. The PI solution was then mixed again and observed via microscopy. The change in turbidity was calculated from the difference in the optical density (ΔOD_600_) of a sample before and after mixing.

- ΔOD_600_ = OD_600_ (after) − OD_600_ (before).
- Turbidity change = ΔOD_600_/OD_600_ (before).

#### 3.3.3. Bacterial Separation

Each bacterial suspension was mixed with a probe solution, and a bacterial recognition protocol was conducted before separation. The sample was then removed by a syringe and filtered by using a 10 μm Omnipore membrane. The resultant filtrate solution and the filter were observed separately via a microscope.

## 4. Conclusions

Bacterial separation was reported by using the benzoxaborole-based dendrimer probe BenzoB-PAMAM(+), which selectively recognized Gram-positive bacteria. BenzoB-PAMAM(+) was newly synthesized by the condensation of carboxy-benzoxaborole and the PAMAM(G4) dendrimer, and was found to recognize the bacterial saccharide that is part of LTA on a Gram-positive bacterial surface over a wide pH range with the help of an electrostatic interaction. The benzoxaborole recognition site showed a desirable low p*K*_a_ value, and might thus result in good selectivity. BenzoB-PAMAM(+) led to the development of large aggregations of Gram-positive bacteria, whereas aggregation was either not observed or minute in size (>10 μm) for Gram-negative bacteria. The selectivity and size of the Gram-positive bacterial aggregations enabled separation by using a 10 μm membrane filter. The presence of Gram-negative bacteria in the filtrate solution was also confirmed. It is true that a small number of Gram-negative bacteria were involved in the aggregation, but most Gram-negative bacteria were successfully separated by filtration. The collection efficacy for each bacterial species needs to be investigated in future studies.

In summary, BenzoB-PAMAM(+), which was designed as a novel aggregation-based and metal-free separation method, successfully recognized and collected Gram-positive bacteria, demonstrating its potential for application in bacterial separation and concentration from environmental soil or water. We believe that these findings contribute significantly to the study of AMR bacterial distributions in the environment.

## Data Availability

Data sharing not applicable.

## Supplementary Materials

The following are available online at https://www.mdpi.com/article/10.3390/molecules28041704/s1, Figure S1: Aggregate formation at a pH of 7.4, adjusted with PBS. Figure S2: Aggregate formation under excessive amounts of bacterial solution at a pH of 7.4, adjusted with PBS. Figure S3: Aggregate formation under a low concentration of bacterial solution at a pH of 7.4, adjusted with PBS. Figure S4: Microscope images of *S. aureus* ATCC25923 (stained with DAPI) and *E. coli* ATCC25922 (stained with EB) at a pH of 7.4 adjusted with PBS. Figure S5: ^1^H NMR spectrum of compound **2**. Figure S6: ^1^H NMR spectrum of compound **3**. Figure S7: ^1^H NMR spectrum of compound **4**. Figure S8: ^1^H NMR spectrum of BenzoB-PAMAM(+). Figure S9: ^1^H NMR spectrum of compound **5**. Figure S10: ^13^C NMR spectrum of compound **5**. Figure S11: ^1^H NMR spectrum of BenzoB-PAMAM(−).
