# Supplementary material for: Aggregation-Based Bacterial Separation with Gram-Positive Selectivity by Using a Benzoxaborole-Modified Dendrimer
Source: Molecules. 2023 Feb 10;28(4):1704. doi: 10.3390/molecules28041704 (PMC9958924; doi:10.3390/molecules28041704)
Supplement: Supplementary file 1 [file molecules-28-01704-s001.zip › molecules-2181035-supplementary.pdf]

Aggregation-Based Bacterial Separation with Gram-Positive Selectivity by using a Benzoxaborole-Modified Dendrimer

Ayame Mikagi [1,†], Yotaro Takahashi [1,†], Nobuyuki Kanzawa [1], Yota Suzuki [1], Yuji Tsuchido [1,2], Takeshi Hashimoto [1] and Takashi Hayashita [1,*]

1. Department of Materials and Life Sciences, Faculty of Science and Technology, Sophia University, 7-1 Kioi-cho, Chiyoda-ku, Tokyo 102-8554, Japan

2. Department of Life Science and Medical Bioscience, School of Advanced Science and Engineering, Waseda University (TWIns), 2-2 Wakamatsu-cho, Shinjuku-ku, Tokyo 162-8480, Japan

[†]These authors contributed equally to this work

*Corresponding author. E-mail address: ta-hayas@sophia.ac.jp (T. Hayashita).

Contents

S1–S4: Analytical Data

S5–S11: ^{1}H and ^{13}C NMR Spectra

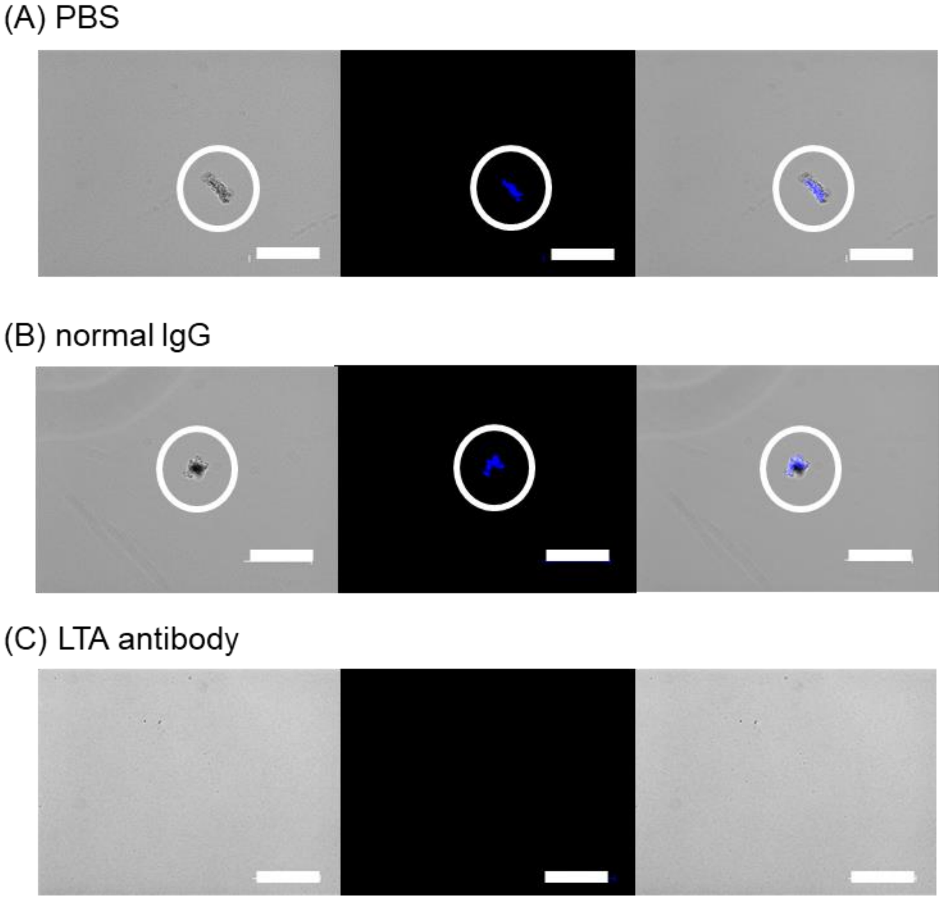

Figure S1. Aggregate formation at pH 7.4 adjusted with PBS. [BenzoB-PAMAM(+)] = 3.3×10^{-6} M, [*S. aureus* IAM1011] = 2.3×10^{6} CFU·mL^{-1}. (A) PBS was used as control. (B) Normal IgG antibody was used as control. (C) LTA antibody, an IgG monoclonal antibody which selectively binds to LTA and disturbs probe's LTA recognition. From left to right: DIC, DAPI, and merged images. Aggregations are shown by white circles. Scale bar = 100 μm. The protocol has already been reported* in our previous article.*

*Mikagi, A.; Manita, K.; Tsuchido, Y.; Kanzawa, N.; Hashimoto, T.; Hayashita, T. Boronic Acid-Based Dendrimers with Various Surface Properties for Bacterial Recognition with Adjustable Selectivity. *ACS Appl. Bio Mater.* **2022**, *5*, 5255–5263. doi: 10.1021/acsabm.2c00680.

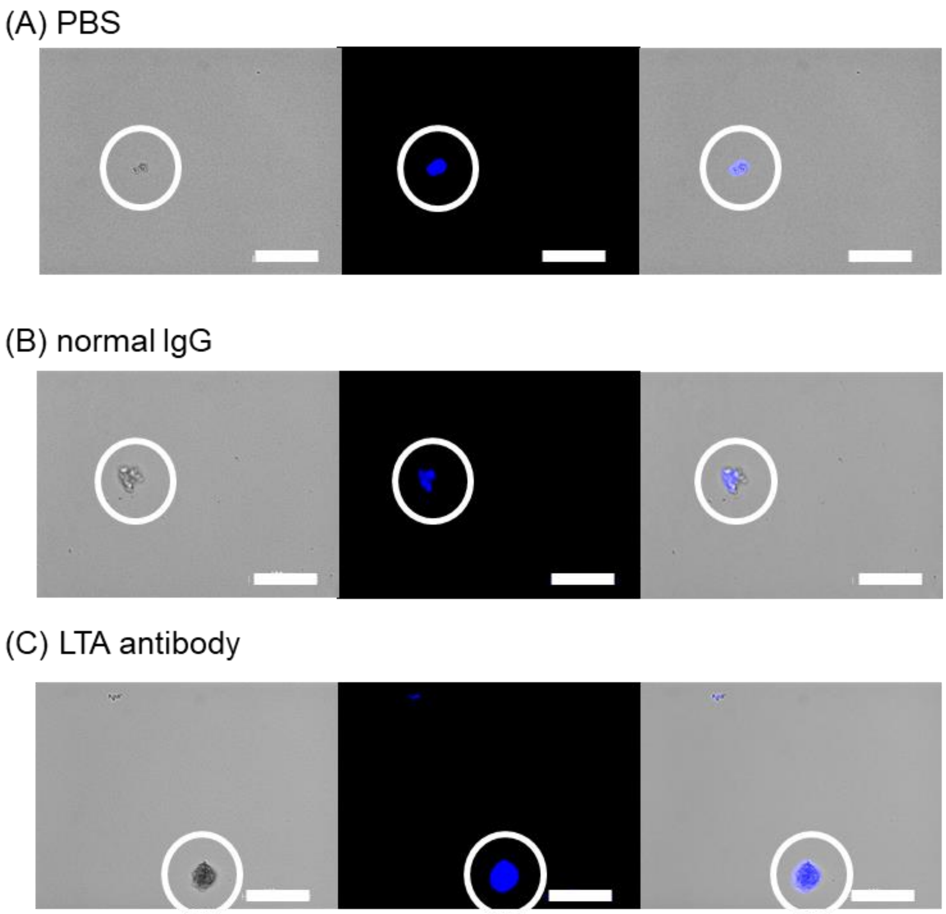

Figure S2. Aggregate formation under excessive amounts of bacterial solution at pH 7.4 adjusted with PBS. [BenzoB-PAMAM(+)] = 3.3×10^{-6} M, [*S. aureus* IAM1011] = 2.3×10^{7} CFU·mL^{-1}. (A) PBS was used as control. (B) Normal IgG antibody was used as control. (C) LTA antibody, an IgG monoclonal antibody which selectively binds to LTA and disturbs probe's LTA recognition. From left to right: DIC, DAPI, and merged images. Aggregations are shown by white circles. Scale bar = 100 μm. Since the excessive amount of bacteria was used, the LTA antibody suspension (C) could not hinder all LTA anymore and aggregation was observed as well as (A) and (B). The protocol has already reported in our previous article.*

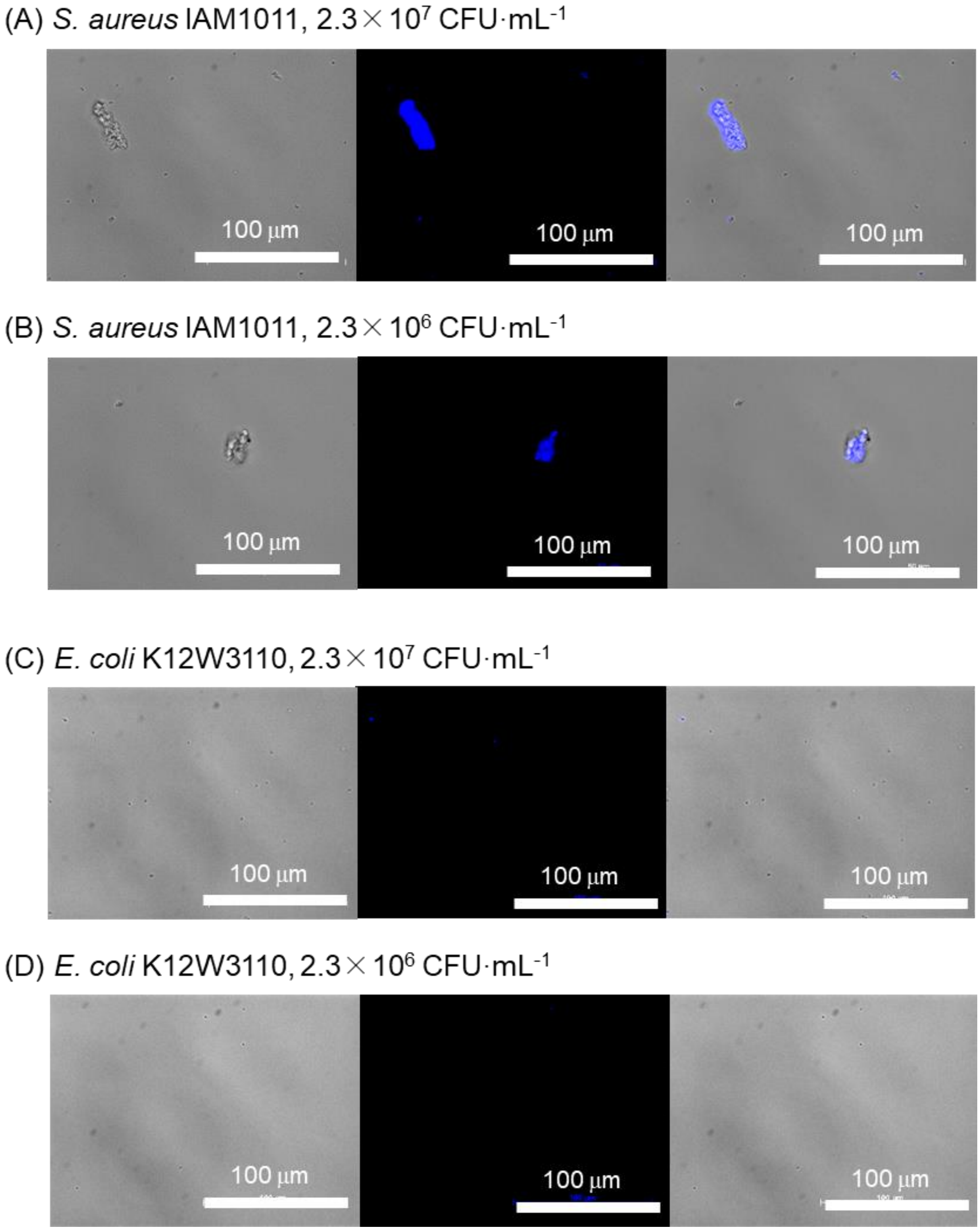

Figure S3. Aggregate formation under low concentration of bacterial solution at pH 7.4 adjusted with PBS. [BenzoB-PAMAM(+)] = 3.3×10^{-6} M. From left to right: DIC, DAPI, and merged images. Scale bar = 100 μm. (A,B) Aggregation was observed. (C,D) Aggregation was not observed.

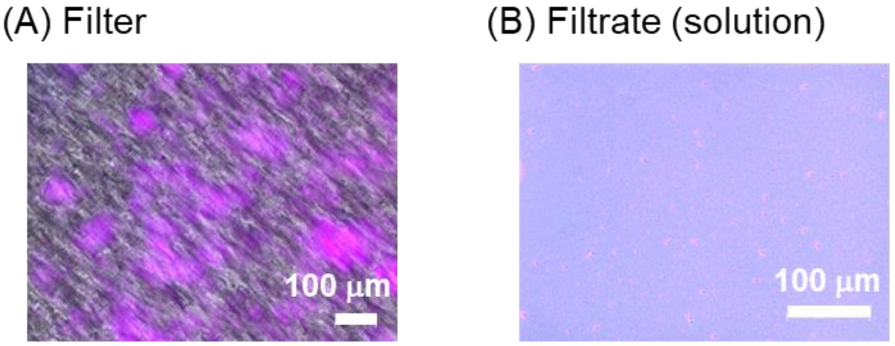

Figure S4. Microscope images using *S. aureus* ATCC25923 (stained with DAPI) and *E. coli* ATCC25922 (stained with EB) at pH 7.4 adjusted with PBS. [BenzoB-PAMAM(+)] = 3.3×10^{-6} M, bacterial concentration was set at OD_{600} = 0.3. The sample's merged microscopy images. Scale bar =100 μm. The image of (A) depicts large aggregation whereas (B) shows dispersed bacteria stained with EB.

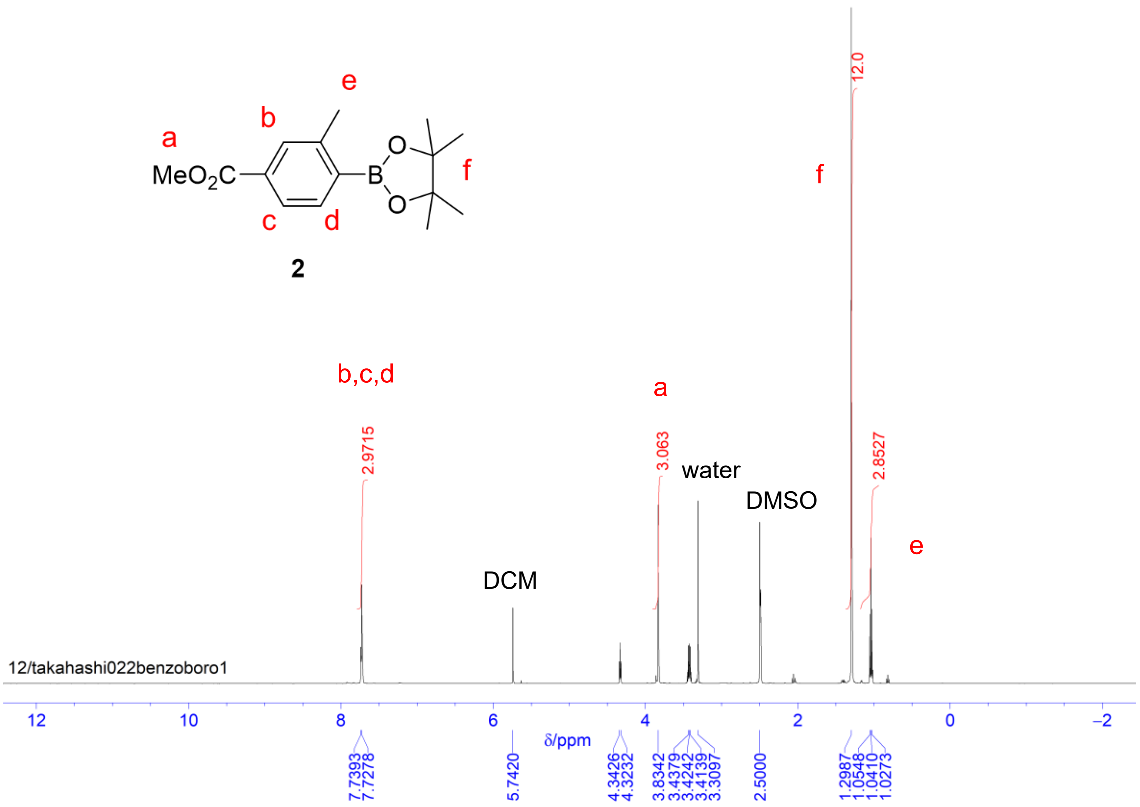

^{1}H NMR (500 MHz, DMSO-d_6) δ(ppm): 1.04 (m, 3H, H_e), 1.30 (s, 12H, H_f), 3.83 (s, 3H, H_a), 7.73 (m, 3H, $H_{b,c,d}$)

*impurities were excluded before the final product **4**.

Figure S5. ^{1}H NMR spectrum of compound **2**.

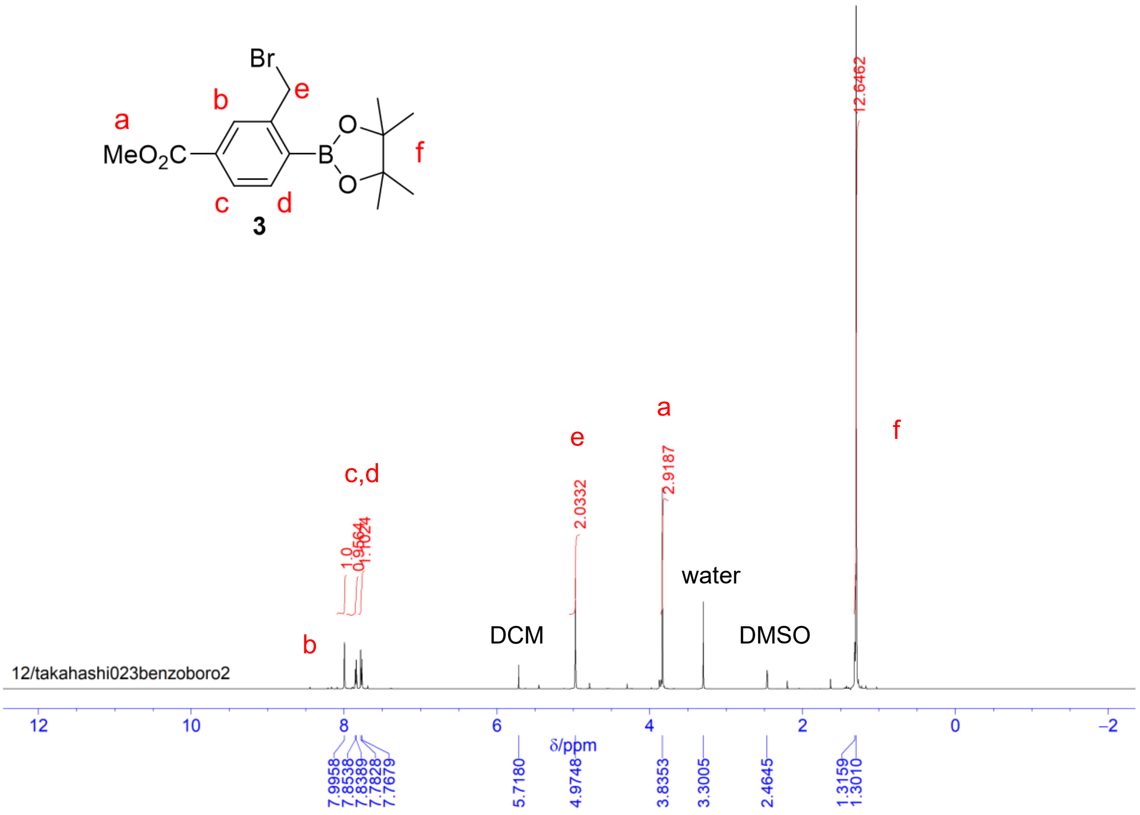

^{1}H NMR (500 MHz, DMSO-d_6) δ(ppm): 1.30 (s, 12H, H_f), 3.83 (s, 3H, H_a), 4.97 (s, 2H, H_e), 7.77 (d, *J* = 7.5 Hz, 1H, H_d) 7.84 (d, *J* = 7.5 Hz, 1H, H_c) 8.00 (s, 1H, H_b)

Figure S6. ^{1}H NMR spectrum of compound **3**.

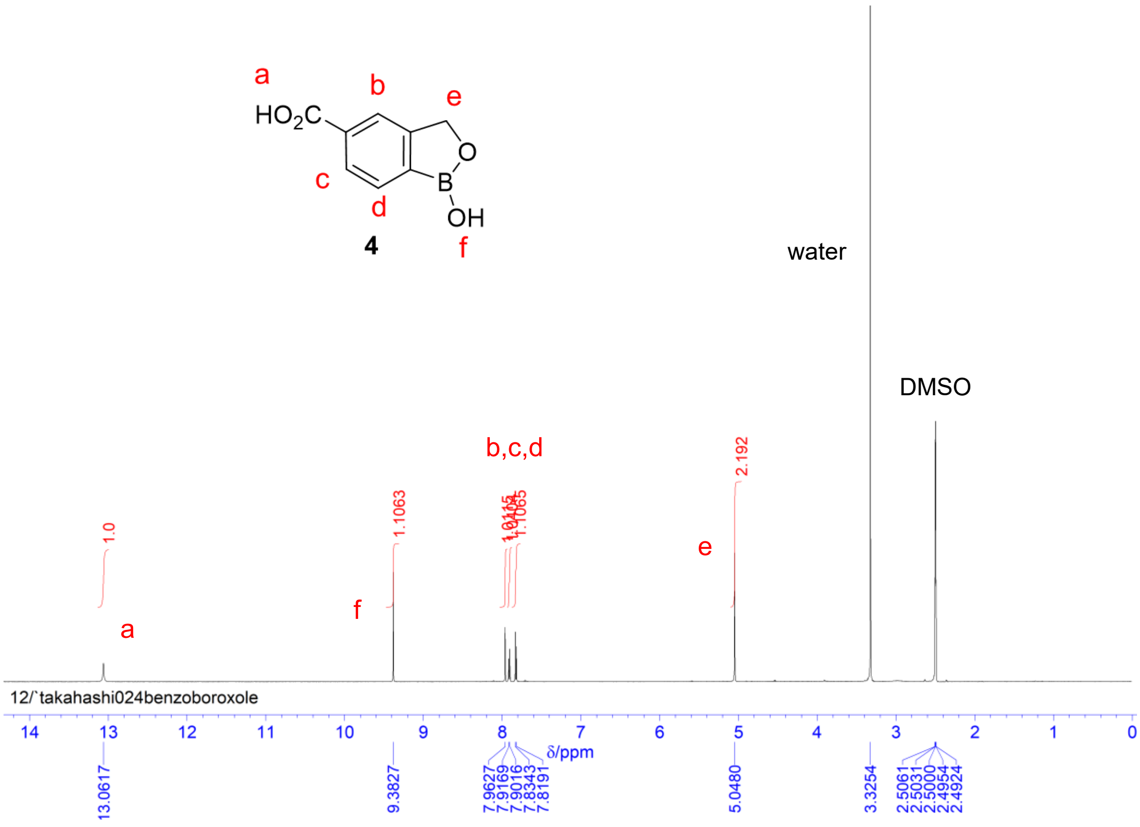

^{1}H NMR (500 MHz, DMSO-d_6) δ(ppm): 5.05 (s, 2H, H_e), 7.82 (d, *J* = 7.6 Hz, 1H, H_d) 7.91 (d, *J* = 7.7 Hz, 1H, H_c) 7.96 (s, 1H, H_b), 9.38 (s, 1H, H_f), 13.1 (s, 1H, H_a)

Figure S7. ^{1}H NMR spectrum of compound **4**.

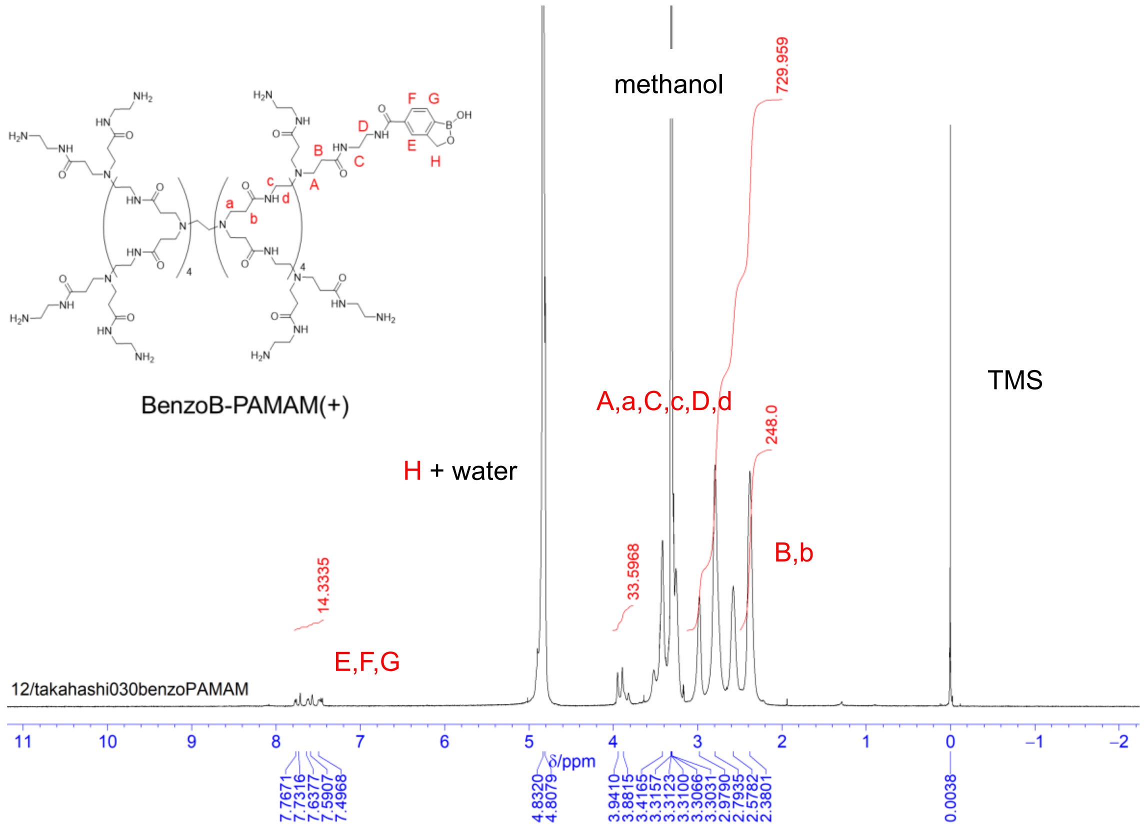

^{1}H NMR (500 MHz, CD_3OD) δ(ppm): 2.38 (br, 248H, $H_{B,b}$), 2.58 (br, 744~753H, $H_{A,a,C,c,D,d}$), 2.79 (br, 744~753H, $H_{A,a,C,c,D,d}$), 2.98 (br, 744~753H, $H_{A,a,C,c,D,d}$), 3.30 (br, 744~753H, $H_{A,a,C,c,D,d,}$), 3.32 (br, 744~753H, $H_{A,a,C,c,D,d}$), 3.42 (br, 744~753H, $H_{A,a,C,c,D,d}$), 3.88 (br, 744~753H, $H_{A,a,C,c,D,d}$), 4.81–4.83 (s, (2X)H, H_H and D_2O), 7.50–7.77 (m, (3X)H, $H_{E,F,G}$),

(E+F+G):(B+b) = (3X):248 (X: The number of modified benzoxaborole)

14.33:248 = (3X):248

M = 4.8

Figure S8. ^{1}H NMR spectrum of BenzoB-PAMAM(+).

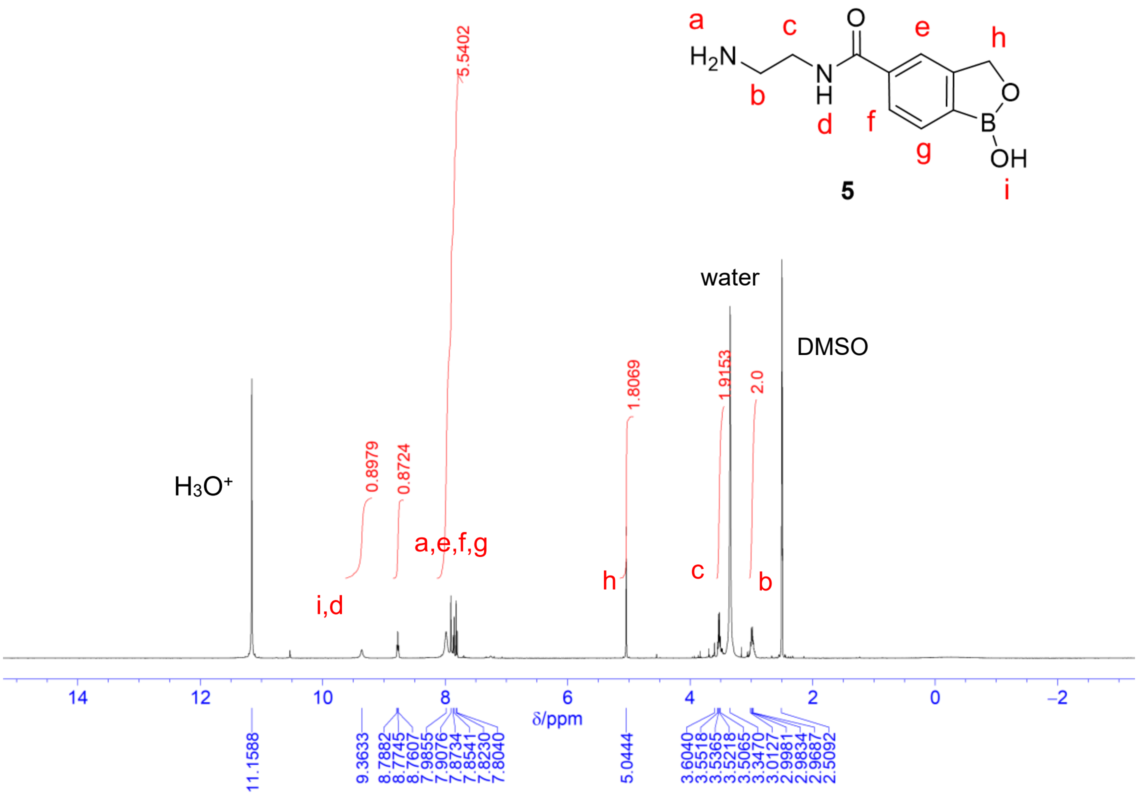

^{1}H NMR (400 MHz, DMSO-d$_6$) δ(ppm): 2.98 (m, 2H, H$_b$), 3.53 (m, 2H, H$_c$), 5.04 (s, 2H, H$_h$), 7.80–7.99 (m, 5H, H$_{a,e,f,g}$) 8.77 (t, J = 6.9 Hz, 1H, H$_d$) 9.36 (s, 1H, H$_i$)

Figure S9. ^{1}H NMR spectrum of compound **5**.

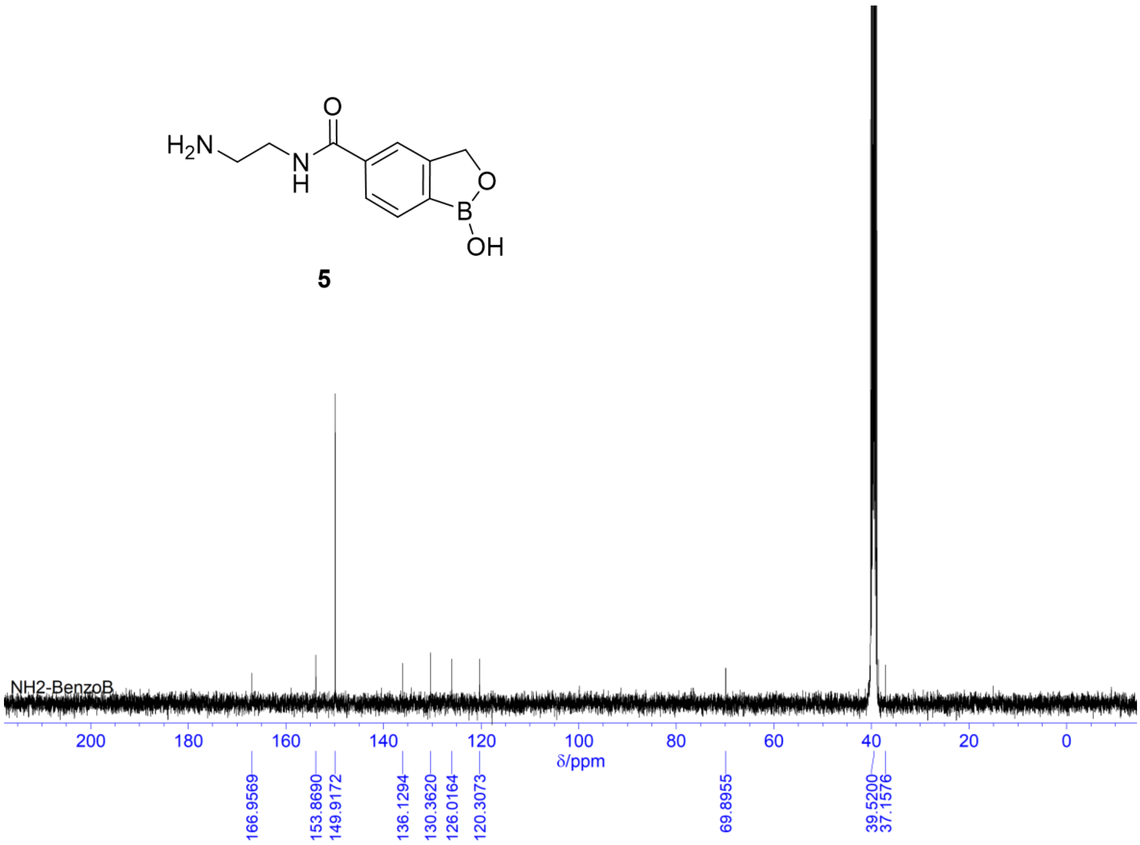

^{13}C NMR (100 MHz, DMSO-d_6) δ(ppm): 37.2, 69.9, 120.3, 126.0, 130.4, 136.1, 149.1, 153.9, 167.0

Figure S10. ^{13}C NMR spectrum of compound **5**.

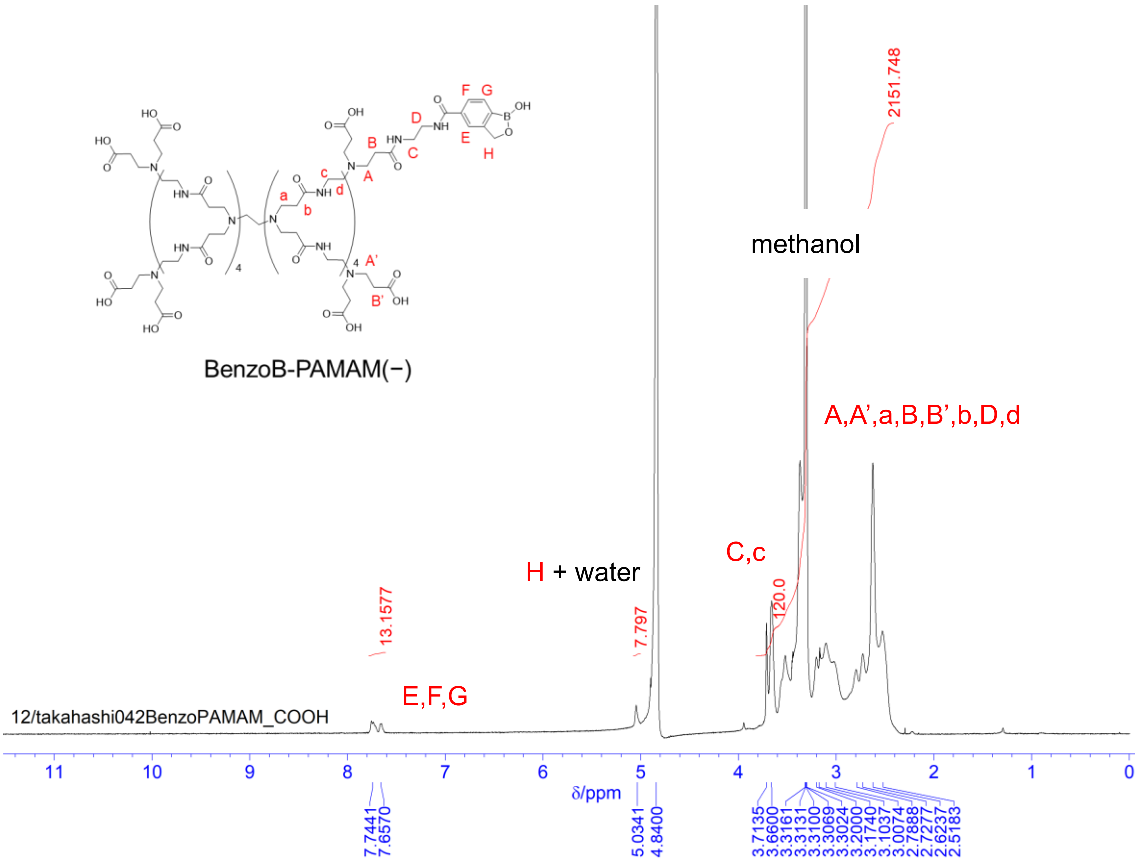

^{1}H NMR (400 MHz, CD_3OD) δ(ppm): 7.80 (m, (2X)H, H_E), 7.72 (m, (2X)H, H_F), 2.52–2.78 (br, (2X+616)H, $H_{A,A',a,B,B',b,D,d}$), 3.00–3.32 (br, (2X+616)H, $H_{A,A',a,B,B',b,D,d}$), 3.66–3.71 (br, (2X+120)H, $H_{C,c}$), 5.03 (s, (2X)H, H_H), 7.66 (m, (3X)H, $H_{E,F,G}$), 7.74 (m, (3X)H, $H_{E,F,G}$)

(E,F,G):(C,c) = (3X):(2X+120) (X: The number of modified benzoxaborole)

13.16:120 = (3X):(2X+120)

X = 4.7

Figure S11. ^{1}H NMR spectrum of BenzoB-PAMAM(−).
